# Response to primary canine core vaccination in 10-month-old seronegative dogs treated with three times the recommended therapeutic dose of Ilunocitinib tablets (Zenrelia™)

**DOI:** 10.1186/s12917-025-04896-5

**Published:** 2025-07-14

**Authors:** Genevieve M. Fent, Simona Despa, Les Gabor, Madison Earll, Erin E. McCandless, Sandra O’Kelley, Jared R. Patch, Jonathan Snyder, Stephen King

**Affiliations:** 1 Elanco Animal Health, Greenfield, IN USA; 2 Elanco Animal Health, Yarrandoo R&D Centre, Kemps Creek, 2178 Australia; 3 Erin McCandless Consulting LLC, Dexter, MI USA

**Keywords:** JAK inhibitor, Vaccination, Ilunocitinib, Zenrelia, Coccidiosis, Immune response, Canine adenovirus type-1

## Abstract

**Background:**

This study was designed to test the effect of a novel JAK inhibitor, ilunocitinib, on the response to modified live and inactivated vaccines when given at three times the label dose to healthy, vaccine-naive, seronegative, juvenile (10-month-old) purpose-bred research dogs. During the study, an outbreak of infectious diseases occurred including coccidiosis and a confirmed case of infectious canine hepatitis. Although confounded by disease, the data provides information to researchers and clinicians on a new member of this important class of therapeutics.

**Results:**

Opportunistic infection by *Cystoisospora canis* was confirmed during the study and associated with mild to severe gastrointestinal disease. Morbidity was higher in the treated group compared to placebo controls and two treated dogs were humanely euthanized due to deterioration of overall health status. Decreased body condition and impaired CD4 T helper cell counts correlated with disease severity. After study completion, Canine Adenovirus Type-1 was identified via PCR in one dog. Despite an outbreak of concurrent disease, all dogs successfully achieved threshold titers to multivalent modified live vaccination administered on day 28. On day 88, responses to modified live booster and inactivated primary rabies vaccinations were decreased where 2/6 treated dogs versus all controls successfully responded to rabies vaccination. Lack of response was correlated to individuals with the most prominent clinical disease and lowest CD4 T helper cell counts. At the end of the treatment phase (day 88), all dogs recovered from disease and most demonstrated improved rabies titer levels by day 116.

**Conclusion:**

Considering the mechanism of JAK inhibitors, it is not surprising dogs receiving three times the expected dose of drug had higher incidence of parasitic and viral disease compared to controls. In the face of these confounding infectious diseases, it was also not surprising that treated dogs had lower measures of health status correlating directly to both disease severity and inactivated vaccine response. Although confounding disease prevents a definitive conclusion regarding the original intent of the study, it was interesting that there was sufficient threshold response relating to the initial modified live vaccination across both control and Jak inhibitor treated groups administered on day 28, however this was not the primary endpoint of the study.

**Supplementary Information:**

The online version contains supplementary material available at 10.1186/s12917-025-04896-5.

## Background

Ilunocitinib is a non-selective Janus-Kinase (JAK) inhibitor that prevents the function of a variety of pruritogenic, pro-inflammatory and allergy related cytokines (IL-2, IL-4, IL-6, IL-13, IL-31 and IFN-g) which depend on the JAK family of enzymes for expression. Ilunocitinib has a high potency for inhibition of JAK1, JAK2, and tyrosine kinase 2 (TYK2) and has been developed as a treatment for allergic and atopic dermatitis (AD) in dogs one year of age or older [1].

Atopic dermatitis is a complex clinical syndrome of pruritus, skin inflammation, and secondary infection affecting up to 15% of the canine population [2]. Currently, understanding of the pathobiological mechanisms of AD is incomplete, but dysregulation of inflammation including cytokines and T cell activity is a hallmark feature of this immune mediated disease [3]. JAK inhibitors have been safely and efficaciously used to treat multiple inflammatory diseases, including AD, in humans [4] and specifically AD in dogs for over 10 years [5]. The JAK family of proteins, expressed on a wide variety of cell types including lymphocytes, are critical mediators of cytokine signaling, and drive inflammatory processes [6]. Currently there are eleven JAK inhibitors approved for use in humans and one for dogs. Oclacitinib is a non-selective JAK inhibitor (JAK1 and JAK3 inhibitor) compound that suppresses proinflammatory cytokines associated with canine AD pathobiology including IL-2, IL-4, IL-6, IL-13, and IL-31 [7, 8] and was approved for use in dogs by the U.S. Food and Drug Administration, Center of Veterinary Medicine in 2013. Expansion of the JAK inhibitor class of therapeutics for dogs provides veterinarians with additional options and broadens their toolkit to meet the unique needs of individual patients.

Overlap exists between the immunological mechanisms that are believed to contribute to the efficacy of immune-modulating therapeutics, such as JAK inhibitors, and those that drive the protective immune response elicited by vaccines. Although vaccine protective mechanisms are not fully understood and differ for various types of vaccines, the antibody- and cell-mediated responses involved utilize the JAK family of signal transduction proteins and associated cytokines [9]. Therefore, it warrants that the effect of JAK inhibition during vaccination be tested. To accomplish this, a study was designed to test the response to primary vaccination with modified live (ML) and inactivated rabies vaccines using a conservative study design to ensure visibility of any treatment-related effect on vaccine response. Differences between the typical patient population and this study included drug treatment at three times (3X) the therapeutic dose of ilunocitinib and administration to barrier-raised, vaccine-naïve, seronegative, purpose bred research animals.

During this study, an outbreak of infectious diseases occurred including cases of coccidiosis (*Cystoisospora canis*) and a confirmed case of infectious canine hepatitis (Canine Adenovirus Type-1). This outbreak severely confounded the study making definitive conclusions according to the original experimental design challenging. As a result, the data presented here will instead describe the effect of a 3X dosing regimen of a JAK inhibitor on the response to ML and inactivated vaccination in unhealthy dogs concurrently with infectious disease. While not the intended purpose of the study, the data generated holds value to share and provide additional information to researchers and clinicians regarding this important class of therapeutics for canine AD.

## Methods

### Objectives and standards

The study objective was to evaluate the impact of orally administered ilunocitinib tablets on the immune response to primary vaccination [canine distemper virus (CDV), canine parvovirus (CPV), canine adenovirus-2 (CAV-2), canine parainfluenza virus (CPiV), and rabies] in dogs.

The animal phase of this study was conducted at a contract research laboratory (CRL) in the United State in accordance with Good Laboratory Practice (GLP). The protocol was reviewed, and conduct of the study approved, by the CRL Institutional Animal Care and Use Committee prior to study initiation. All protocol amendments impacting animal welfare were also approved by the CRL Institutional Animal Care and Use Committee. It was executed in compliance with the US Code of Federal Regulations Title 9, Parts 1–4, The Animal Welfare Regulations; US Code of Federal Regulations Title 7, Chap. 54, Sects. 2131–2159, The Animal Welfare Act and followed the Guide for the Care and Use of Laboratory Animals. The molecular diagnostics described herein were not conducted as part of the original animal study. Molecular diagnostic tests were developed and conducted non-GLP in the laboratory on formalin fixed paraffin embedded (FFPE) tissue generated during the study live-phase.

### Animals and maintenance

A total of 20 (10 males and 10 females) purpose bred Beagle dogs seronegative for CAV-2, CDV, CPiV, CPV and rabies were purchased from a commercial laboratory animal supplier and maintained in a biosecure facility until approximately nine months of age. At this time, all dogs were transferred by road to a second biosecure facility for the in-life phase of the study. All animals were acclimatized at the in-life study site for 21 days before randomization. Sixteen healthy dogs (8 males and 8 females), seronegative for CAV, CDV, CPiV, CPV, and Rabies on Day − 21, were included in the study.

Dogs were pair housed by sex within pens in a single indoor Animal Biosafety Level 2 (ABSL2) room. After randomization, dogs were pair housed by treatment group and sex. Dogs were separated each day for feeding, dosing, and post-dose observations during the dosing period (study Days 0–88). A photo cycle of 12 h light: 12 h dark was maintained. Animal room temperature was maintained between 60.44 and 82.94 °F and relative humidity was between 30 and 70%, although brief excursions were allowed. Each pen was supplied with food bowls, water bowls and enrichment toys.

Dogs had *ad libitum* access to potable water, were fed once daily, and had access to food for four hours. During the dosing period each dog was fasted for at least 16 h, then given 25% of the daily recommended intake of a canned canine diet (Purina Dog Chow High Protein (Classic Ground Beef), Nestlé Purina Pet Care, St. Louis, MO, USA) 20–30 min before dosing. Dogs were allowed 20 min to consume the canned diet. Placebo or ilunocitinib was administered 20–30 min post feeding. After dosing, dogs were given their daily ration of food.

### Allocation to groups

This was a randomized, masked, placebo-controlled study with parallel-group design. On Day − 1, sixteen eligible dogs were randomized, by sex, into either the placebo group (Group 1) or the ilunocitinib-treated group (Group 2). The randomization procedure was performed using SAS v9.4 (SAS Institute Inc., Cary, NC, USA). Similarly, dogs were randomly assigned to pair housed cages by sex and treatment. The dogs were identified through microchip, and when necessary, a unique ear tattoo was used as a secondary identification.

### Investigational veterinary product, and placebo administration

Group 1 dogs received placebo tablets, while Group 2 received ilunocitinib tablets orally at 2.4 mg/kg (3X) daily followed by a water flush from Day 0 to 88. The number of placebo tablets given was consistent and matched the highest tablet exposure level in dogs treated with ilunocitinib. The ilunocitinib dose was based on the most recent weekly weight measurement and rounded up to the next half tablet to ensure a minimum dose of 2.4 mg/kg. After dosing, the dog’s mouth was inspected to confirm the tablet was swallowed. If a dog vomited or expelled a tablet within 2 h, it was re-dosed once. If the expelled dose was recognizable and able to be handled, it was re-administered.

### Vaccines and vaccination

Commercially purchased vaccines, Nobivac^®^ Canine 1-DAPPV (Merck Animal Health, Rahway, NJ, USA) and IMRAB^®^ 3 (Boehringer Ingelheim, Ridgefield, CT, USA), were administered as per label directions provided on the package inserts. The Nobivac^®^ Canine 1-DAPPV vaccine was administered on Day 28, and both Nobivac^®^ Canine 1-DAPPV and IMRAB^®^ 3 vaccines were administered on Day 60, which was delayed from Day 56 to allow study animals impacted by an unexpected disease outbreak additional recovery time.

### Assessments and variables recorded

Animals were observed at least twice daily throughout the study. Observations included general behavior, body systems, feces (color, consistency, presence of blood and mucus), locomotion, and other symptoms such as abnormal urine, salivation, vomiting, and presence of blood.

Body weights were recorded initially on Day − 21 and Day − 2, and then approximately weekly thereafter. Food intake was measured daily throughout the study.

Blood and urine were collected for clinical pathologic evaluation (clinical chemistry, complete blood count (CBC), coagulation, urinalysis) on Days − 21, -7, 28, 88, and 172. All samples were sent to Antech GLP, USA for analysis. Blood samples in EDTA tubes were analyzed for CBC using the Advia 120 and 2120i automated hematology systems (Siemens, Malver, PA, USA). Serum samples were evaluated via Coulter AU680 Clinical Chemistry Analyzer (Beckman Coulter, Brea, CA, USA). Plasma samples were tested using the CS-2500 Coagulation Analyzer (Sysmex, Lincolnshire, IL, USA). Urinalysis was performed using the Multistix 10SG method on the Clinitek^®^ Advantus Urine Chemistry Analyzer (Siemens, Malver, PA, USA).

### Fecal examinations

Qualitative fecal examinations were conducted on all dogs during acclimation (Day − 10 or Day − 9) to check for parasites. Additional examinations were performed on Days 26, 28, 35, 42, 56, 57, and 58 due to persistent diarrhea, weight loss, and lack of appetite in some study animals.

### Immunophenotyping

Blood samples collected in K_2_EDTA tubes on Days − 21, -7, 28, and 88 were used for immunophenotyping (IMT). All processing, staining, and flow cytometric procedures including data analysis were carried out at Charles River Laboratories, Ashland, Ohio, USA according to their validated methods. Results were acquired with a FACSCanto II flow cytometer (BD Biosciences, Franklin Lakes, NJ, USA) and analyzed with FlowJo (Ashland, OR, USA) software. Immunophenotyping antigens and cell populations were measured using the following markers: Total T lymphocytes (CD45 + CD3+), Helper T lymphocytes (CD45 + CD3 + CD4+), Cytotoxic T lymphocytes (CD45 + CD3 + CD8+), B lymphocytes (CD45 + CD5-CD21+), and Monocytes (CD45 + CD3-CD14+).

### Serum titers against vaccine antigens

Antibody titers against CDV, CPiV, and CAV were measured using serum neutralization, and against CPV using hemagglutination inhibition. Rabies antibody titers were determined using the Rapid Fluorescent Focus Inhibition Test (RFFIT). Blood samples (6.0 mL in serum separator tube) for titer determination were collected twice during acclimatization (Days − 21 and − 7) and on Days 28, 60, 88, 116 (rabies titer only), and 172. The study design is shown in Table 1. Samples collected on Days 28, and 60 were prior to vaccination. Serum was separated and frozen prior to shipping. CAV, CDV, CPiV, and CPV antibody titer determination was performed by the Animal Health Diagnostic Center (College of Veterinary Medicine, Cornell University, Ithaca, NY, USA) and rabies titers were performed by Atlanta Health Associates, Inc, Cumming, GA, USA.

**Table 1:** Study design

Study Day	-21	-7	0	28	60	88	116	172
Dose 0 or 2.4 mg/kg (3X)			X-------------------------------------------------------X^+^					
Primary ML Vaccine				X				
Booster ML & Primary Rabies Vaccine					X			
Measure ML and Rabies Titers	X	X		X	X	X	X*	X

*Rabies titer only, ^+^Indicated dosing daily during the time interval

### Pathological examination

Two dogs were humanely euthanized due to deterioration of overall health status via an intravenous overdose of sodium pentobarbital prior to study completion on either Day 52 or 54, and postmortem evaluations were performed. Gross and histopathologic analysis were performed by a Board-Certified Veterinary Pathologist.

### Molecular diagnostics

#### Polymerase chain reaction (PCR) method

For formalin fixed paraffin embedded (FFPE) study samples, the modified method of Vitošević et al. was used to melt and remove the paraffin prior to tissue lysis, reverse-crosslinking, and DNA extraction [10]. Phenol-chloroform extraction and ethanol precipitation was performed on FFPE extracted DNA to clean and concentrate the DNA. Formalin fixed tissue from experimentally CAV-1-infected and non-infected animals was used for controls. Approximately 10–25 mg of tissue was homogenized for nucleic acids extraction using the modified method of Oba et al. for tris-mediated DNA extraction [11].

DNA concentration was measured using a Qubit (ThermoFisher Scientific, Waltham, MA) and normalized by dilution to a target of 50 ng/µl. For the first round of nested PCR, 200 ng of DNA template was amplified using the PrimeSTAR GXL Polymerase kit (Takara Bio, San Jose, CA). For the second round of nested PCR, 1.0 µl of template derived from the first-round product was used. The PCR reaction was performed with the following thermocycler settings: 98 °C for 10 s, 60 °C for 15 s, 68 °C for 5 s, for 35 cycles followed by a hold at 4 °C. PCR products were analyzed by agarose gel electrophoresis in comparison to a DNA ladder standard. DNA bands were excised from agarose gel and DNA purified using a QIAquick Gel Extraction Kit (Qiagen, Germantown, MD). DNA was shipped to ACGT, Inc. (Wheeling, IL) for Sanger sequencing using primer CAV-1_10F: 5’-GGGGGCGTTTGGTTTTTCTGCTCTGT. Low quality base reads were trimmed prior to alignment. Purified DNA was also cloned using a Zero Blunt TOPO PCR Cloning Kit (ThermoFisher, Waltham, MA), according to kit instructions. Twelve resulting colonies were screened by miniprep and subsequent EcoRI digest. Three colonies were shipped to ACGT, Inc. for Sanger sequencing using primers M13 Forward and M13 Reverse. Vector sequence was removed prior to alignment.

#### Oligonucleotide PCR primers

Oligonucleotide PCR primers (Integrated DNA Technologies, Coralville, IA) to detect CAV-1 in tissue samples were adapted from Walker et al. [12]. A new nested PCR strategy was designed where the 188-base pair (bp) fragment generated using the primers of Walker et al., was followed by amplification of a smaller 168 bp fragment using newly designed primers [12]. The second-round primers were designed by alignment of representative CAV-1 and CAV-2 sequences to achieve specificity and are shown in Table 2. A set of primers (designed by Primer3 software using GCF_003254725.2 genome assembly) to the canine actin beta gene were used as a control for total DNA extraction and amplification. Amplification of actin beta was performed with a single, non-nested, PCR reaction.

**Table 2:** PCR primers

Nested Round	Name	Sequence	Target	Expected Amplicon Size (bp)
1	CAV-1_2F	ATTATCGTGTTTAGATGGGGGGC	CAV-1	188
	CAV-1_2R	GTAACAGCCCAGCTAGTTAACAAG	CAV-1	
2	CAV-1_9F	GTTTAGATGGGGGGCGTTTGGT	CAV-1	168
	CAV-1_9R	CTAGTTAACAAGCTTATAGAATCATACC AGATTTTT	CAV-1	
Not Applicable	ACTB-F	ATCTACGAGGGGTACGCCTT	actin beta	190
	ACTB-R	CCATCTCCTGCTCGAAGTCC	actin beta	

## Results

### Animal observations

The test article related abnormal findings were consistent with the expected pharmacology of immune-modulating therapeutics and predominately involved the skin and opportunistic infection. It is important to note that based on the confirmation of confounding infectious disease, clinical and laboratory evaluations described below are not expected to be representative of healthy dogs.

#### Skin

Skin lesions occurred in 50% of control and 87.5% of treated dogs. Lesions consisted primarily of dermal reddening or thickening, and interdigital cysts in both groups as well as ear crusting in some treated animals.

#### Opportunistic infection

An outbreak of mild to severe gastrointestinal (GI) disease occurred during this study beginning around Day 10 and continuing through ~ Day 50. In general, ilunocitinib treated dogs had more severe clinical disease than placebo controls. All ilunocitinib treated dogs had clinical signs of GI related illness (persistent diarrhea, vomiting, weight loss, and inappetence) with an incidence rate of 34% (220 events/641 doses) for vomiting and diarrhea, while six of the eight placebo dogs had clinical signs of GI illness with an incidence rate of 2% (12 events/712 doses) for vomiting and diarrhea. Qualitative fecal flotations were performed on all dogs during the outbreak of diarrhea and dogs with severe GI upset were also tested for CPV and Giardia. Tests for Giardia and CPV were negative, but the fecal flotations revealed *C. canis* in seven of eight ilunocitinib treated animals on day 42. Three of these dogs also tested positive for *C. canis* on earlier fecal flotations performed a week to two weeks prior (initial positive result was on Day 26). All study dogs were treated for *C. canis* infections in addition to supportive therapy as recommended by the Test Site Staff Veterinarian. Initial treatment with sulfadimethoxine began on Day 28. Although dosed per label instructions, sulfadimethoxine therapy was not effective as evidenced by the continual worsening of the clinical condition in many animals and subsequent positive fecal exams (Day 42). As such, sulfadimethoxine was discontinued and ponazuril therapy (50 mg/kg active ingredient) was initiated on Day 49 for all animals resulting in the resolution of vomiting and diarrhea around Day 50. Enrofloxacin was also administered (Day 55–59), per label, to all study animals as a sepsis preventative. Metronidazole, veterinary lactated ringers and 50% dextrose were administered on a case-by-case basis dependent on the clinical condition (e.g., dehydration, weakness).

### Clinical manifestations

**Fig. 1:**
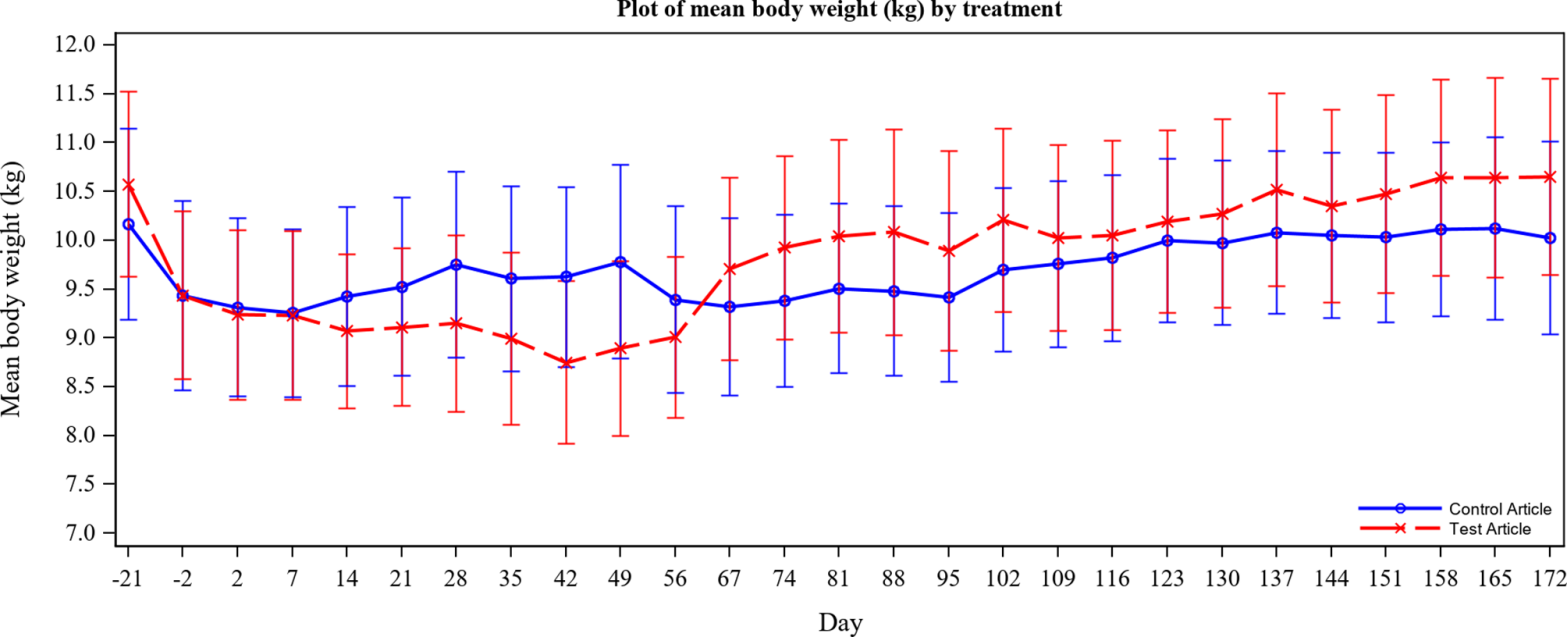
Body Weight. Legend: Mean body weight per group over the duration of the study

All dogs, ilunocitinib and placebo treated, experienced a decrease in body weight during acclimation and the first week of treatment resulting in a diet change. Following the diet change, body weights of the placebo group started to rise, whereas the ilunocitinib treated group continued to experience weight loss through Day 42. The sustained weight loss in the ilunocitinib group is attributed to endoparasitism by *C. canis*. In support of this hypothesis, body weights in the ilunocitinib treated group recovered following effective treatment of the endoparasitism with ponazuril administered on Days 49–55. From Day 67 onwards the average body weight of the ilunocitinib group was higher than that of the placebo group (Fig. 1).

Food consumption in the ilunocitinib treated group decreased between Days 14 to 35, and again from Day 42 to 49. Following this period, food intake fluctuated but generally showed an upward trend for the remainder of the study. At Day 14, the placebo group had higher food intake compared to the treated group, but during the time period between Days 14 and 35, both groups experienced a roughly equal magnitude (approximately 40 g) reduction in food intake. Between Days 42 and 49, both groups continued to decrease food consumption with the ilunocitinib group, achieving a minimum value for the study overall during this time. After Day 49, food intake in the treated group was variable but trending upward through the rest of the study. The control group showed a similar pattern increase throughout the rest of the study with less variability than the treated group. By Day 105, both groups had similar food intake, with the treated group consuming slightly more food than the control group for the remainder of the study.

**Fig. 2:**
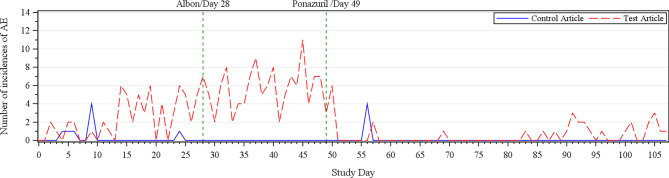
Diarrhea and Emesis Incidence by Day up to Day 106. Legend: Number of incidences of vomiting and diarrhea occurring during the first 105 days of the study. Dotted green lines indicate when treatments for diarrhea began in relation to the incidence of vomiting and diarrhea

The highest incidence of diarrhea and emesis in the treated group roughly corresponded to the timeframe of decreased food intake. By Day 50, corresponding with the start of Ponzuril therapy on Day 49, the incidence of diarrhea and emesis in the treated group dropped dramatically and remained intermittent throughout the completion of the study. A low incidence of intermittent diarrhea or emesis was seen in the control group throughout the study (Fig. 2).

### Clinical pathology

There were reductions in total serum protein, albumin, and globulin in the ilunocitinib treated group on Day 28, but no other discernible trends were observed. The alterations in serum proteins were consistent with protozoal enteritis and related GI protein loss and would be expected in the context of disease resulting from *C. canis* infection. By Day 88, all dogs had total serum protein, albumin, and globulin within normal limits.

### Study removals, gross necropsy and histopathology

Two animals (dog ID 239 and 495) from the ilunocitinib treated group were removed from the study and humanely euthanized due to clinical decline. Necropsies and histopathologic examinations were performed on both dogs. All other animals completed the study.

The presumptive diagnosis following gross necropsy for dog 239 was colitis, ascites, and poor body condition, and for dog 495 fat necrosis with possible pancreatitis was noted. The microscopic examination of dog 239 was unremarkable with significant cold storage artifact present. Microscopic examination of dog 495 revealed marked necrotizing hepatitis, pancreatitis, and the presence of intranuclear inclusion bodies consistent with infectious canine hepatitis (ICH).

### Molecular diagnostics

Due to the presumptive diagnosis of ICH during postmortem evaluation of dog 495, confirmative diagnostic tests were performed to definitively identify the presence of CAV-1. As stated above, this work was not performed as part of the original study but used tissues generated from the in-life phase of the study. PCR is the most sensitive method for the detection of nucleic acids that are derived from the canine adenovirus genome, therefore a PCR method for detection of CAV-1 in FFPE liver tissue was developed and performed.

#### PCR sample analysis

**Fig. 3:**
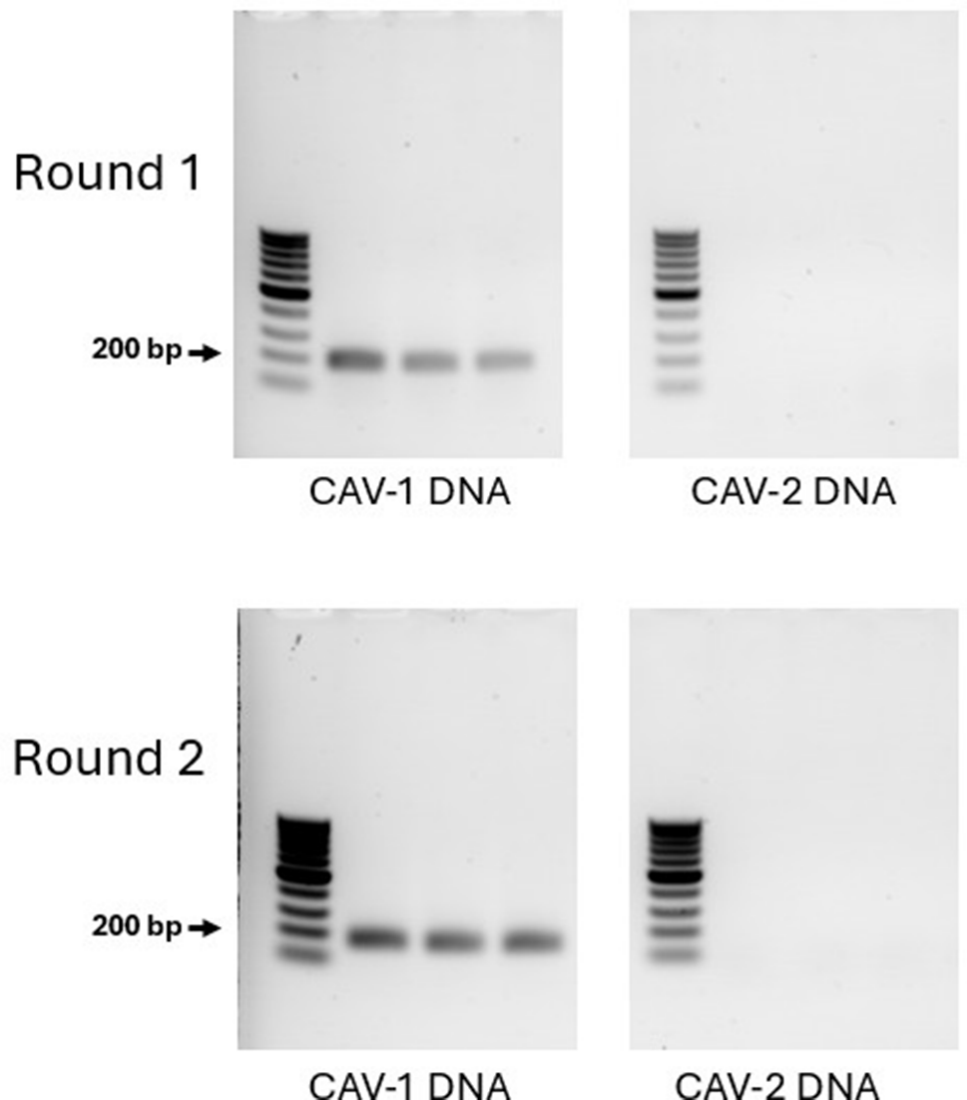
Nested PCR Specificity. Legend: Nested PCR specificity was confirmed in two rounds of PCR using CAV-1 or CAV-2 as a template. In both rounds of PCR using CAV-1 as the template bands were detected, but no bands were detected when using CAV-2 as the template, thus confirming CAV-1 specificity

**Fig. 4:**
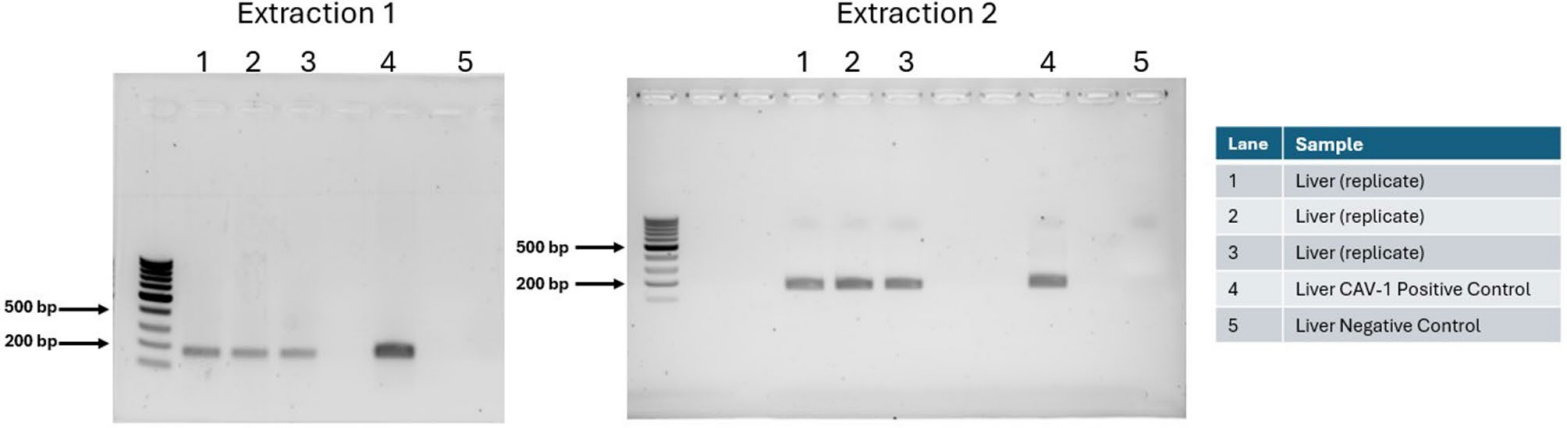
Detection of CAV-1 from Dog 495 FFPE Liver Tissue. Legend: Two independent DNA extractions were tested for the presence of CAV-1 genome by PCR on different days. Both extractions yielded bands consistent with the presence of CAV-1 genome and parallel positive and negative control tissue templates provided supporting evidence the detected bands were CAV-1

To assess the presence of CAV-1 DNA in tissue from Dog 495, DNA was extracted from the FFPE liver sample and subjected to PCR analysis as described in the Materials and Methods (Figs. 3 and 4).

Two independent DNA extractions were tested for the presence of CAV-1 genome by PCR on different days. Both extractions yielded bands consistent with the presence of CAV-1 genome. Parallel positive and negative control tissue templates provided supporting evidence for the authenticity of the detected bands. DNA sequencing was then used to confirm the identity of the PCR bands. Alignment of the CAV-1 and CAV-2 reference genomes (Genbank accessions AC_000003 and AC_000020, respectively), revealed 10 nucleotide differences in the region between the second-round primers for CAV-1. The genomic sequence of CAV-2 in the same vaccine product was previously determined and is identical to the reference genome in this region. None of the CAV-2 sequence differences were found in the PCR products, demonstrating that the PCR amplicon was not derived from the presence of CAV-2 nucleic acids, including the vaccine strain, but rather CAV-1.

### Immunophenotyping

**Fig. 5:**
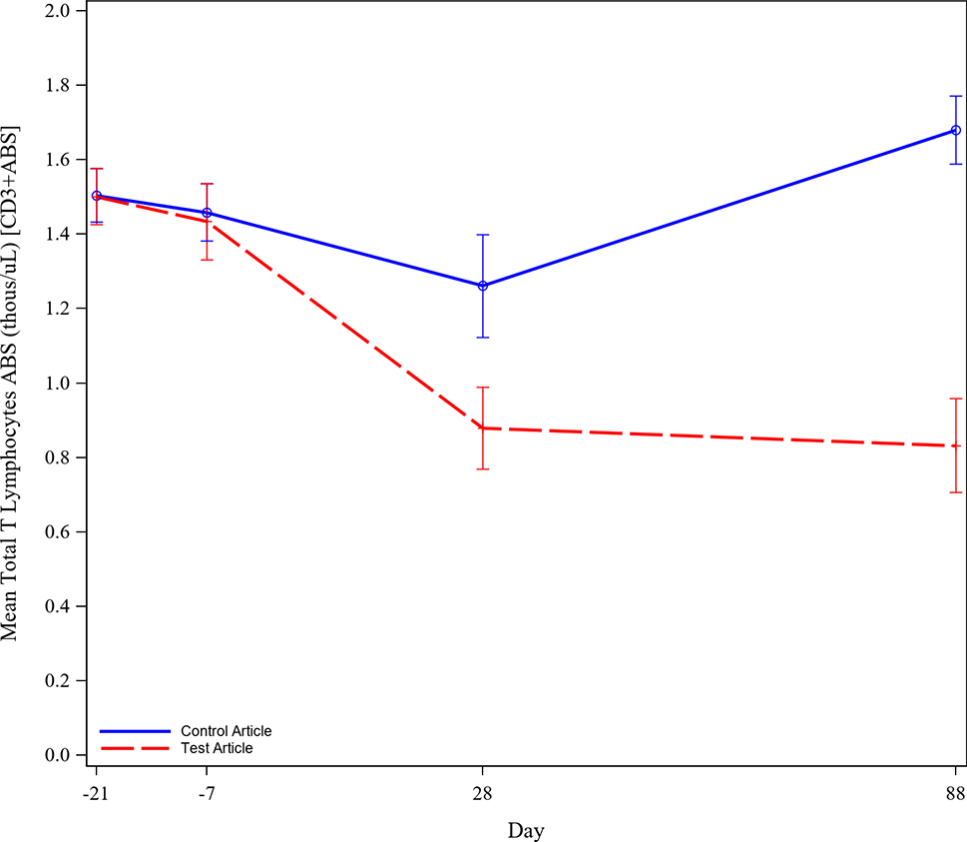
Mean Total T Lymphocytes. Legend: Mean Total T Lymphocytes per group between Day − 21 and 88 of the study

**Fig. 6:**
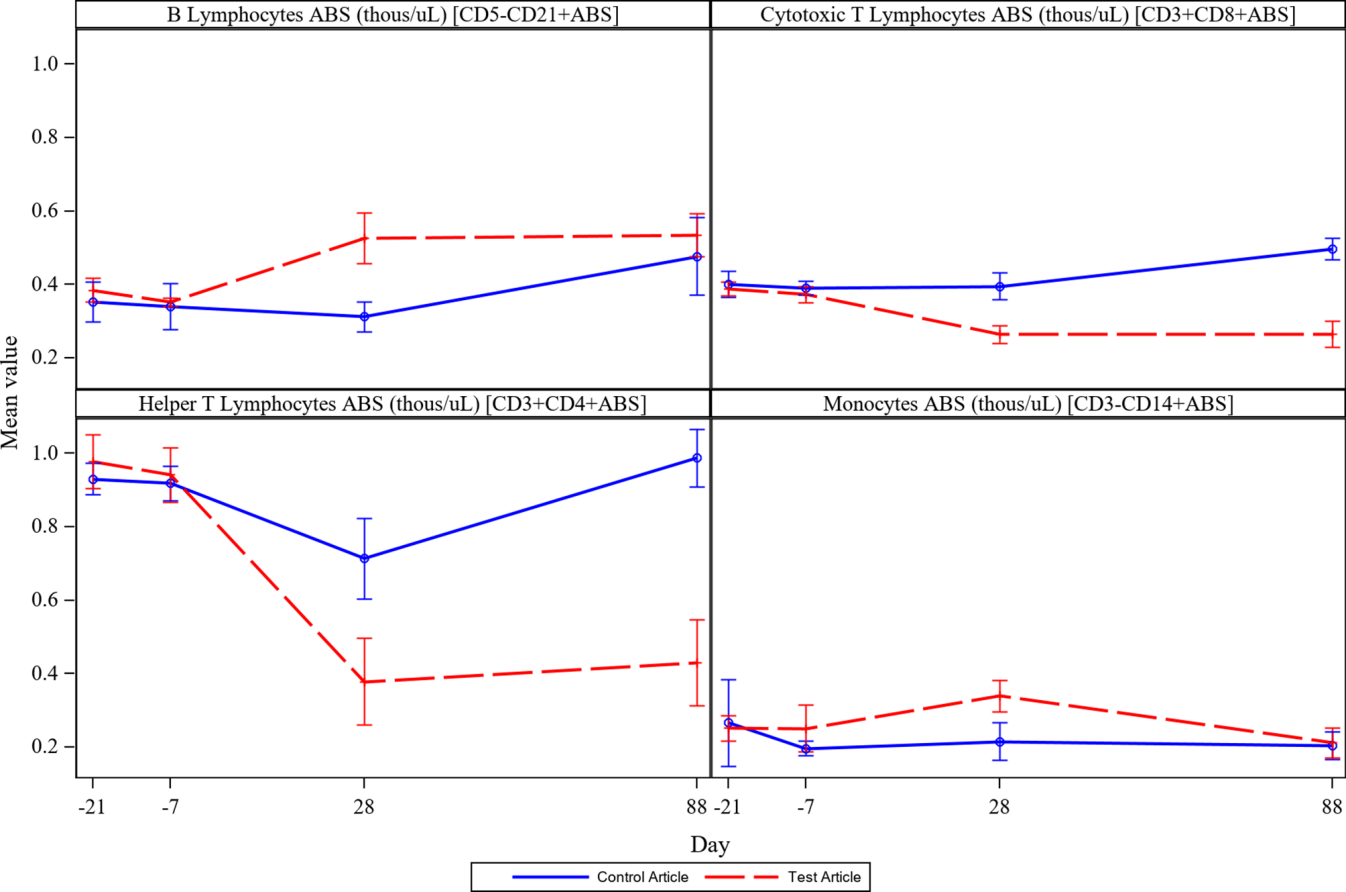
Mean B Lymphocytes, Cytotoxic T Lymphocytes, Helper T Lymphocytes, Monocytes. Legend: Mean B Lymphocytes, Cytotoxic T Lymphocytes, Helper T Lymphocytes, and Monocytes per group between Day − 21 and 88 of the study

Immunophenotyping mean results are shown in Figs. 5 and 6. Five ilunocitinib treated dogs experienced a drop in Th cells compared to baseline on Day 28 which continued through study removal or Day 88, (Fig. 6). Compared to baseline, Day 28 Th cell absolute counts decreased by > 99% in four of these five dogs and decreased 81% in the remaining dog. The dramatic drop in Th cells in these five dogs resulted in absolute Th cell numbers ranging from 0.0 to 0.16 thous/µL on Day 28 and 0.0 to 0.3 thous/µL on Day 88 (reference range: Male-0.67-2.03 thous/µL, Female-0.78-1.8 thous/µL). All but two control dogs also experienced a decrease in Th cells on Day 28 compared to baseline confirming the effect did not result from drug treatment alone. The decrease seen in these dogs was not as prolonged as in the five treated dogs discussed above, and most recovered by Day 60. The Th cell decrease in the control dogs ranged from 6.6 to 63.4% compared to baseline and resulted in absolute Th cell values of 0.3–0.79 thous/µL on Day 28. By Day 88, the absolute Th cell values ranged from 0.61 to 1.25 thous/µL.

### Vaccine responses

Pre-determined titer thresholds (henceforth called threshold) were established prior to study initiation as success criteria for evaluating response to vaccination in healthy dogs. The pre-determined thresholds are shown in Table 3 [13, 14].

**Table 3:** Established titer thresholds to confirm protective immunity

Virus	Analysis performed to determine titer level	Threshold for protective immunity
Canine Distemper Virus (CDV)	Serum Neutralization	≥ 32
Canine Parvovirus (CPV)	Hemagglutination Inhibition	≥ 80
Canine Parainfluenza Virus (CPiV)	Serum Neutralization	≥ 16
Canine Adenovirus (CAV-2)	Serum Neutralization	≥ 16
Rabies	Rapid Fluorescent Focus Inhibition Test (RFFIT)	> 0.5 IU/mL

#### ML vaccines

**Fig. 7:**
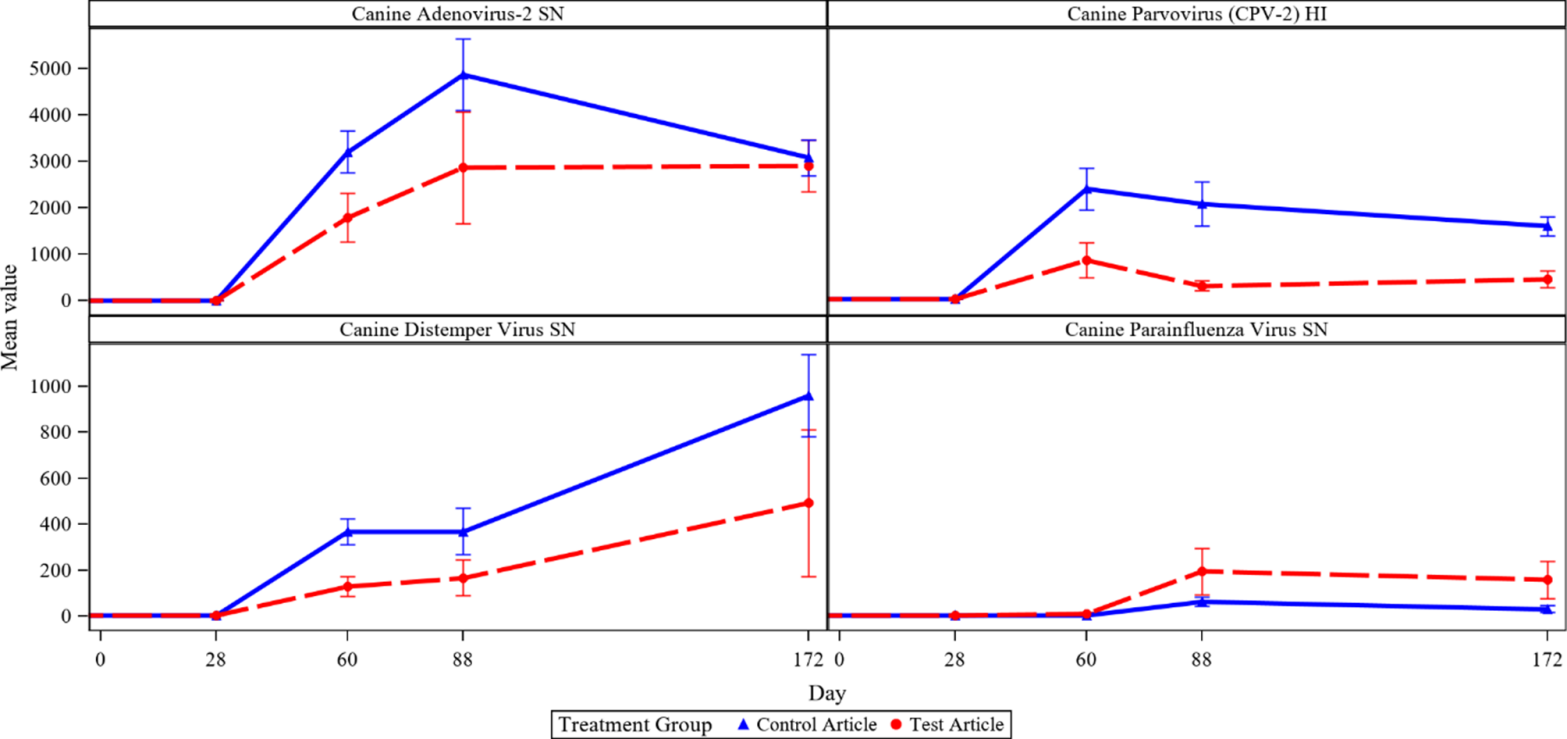
Titer Results for CAV-2, CDV, CPiV and CPV. Legend: Mean Titer results per group for CAV-2, CDV, CPiV, CPV throughout the study

Primary vaccination on Day 28 for CAV-2, CDV, and CPV resulted in all animals responding with titers at or above the threshold for protective immunity on Day 60. This indicates an appropriate response to primary vaccination for these viruses following 60 days of ilunocitinib treatment at 3X the recommended dose (Day 60 titers). For CPiV, the treated group had a higher number of dogs reaching threshold (2 dogs) compared to controls (0 dogs) on Day 60. Overall, ilunocitinib and placebo treated dogs successfully achieved threshold titers to primary vaccination despite the outbreak of clinical disease around the time of vaccination on Day 28. Response to the Day 60 booster was evaluated on Day 88 and all animals responded with titers at or above the threshold for CAV-2 and CPV. All but one ilunocitinib treated dog responded with titers at or above the threshold for CDV on Day 88. The results for CPiV were equivocal between the ilunocitinib and placebo treated groups, with 2 dogs in each group having a titer below threshold on Day 88. Of note is the timing of the booster vaccination, which was delayed by 4-days from the original study design to allow additional recovery time for clinically ill animals resulting from the coccidiosis outbreak. This length of time was deemed sufficient by the Test Site Staff Veterinarian as dogs were no longer experiencing diarrhea and vomiting and were clinically improved. However, the single dog (dog 735) with a titer falling below threshold for CDV on Day 88 was the most clinically impacted by the coccidiosis infection and was still recovering body condition at the time of Day 60 booster vaccination. Based on threshold titer data to primary vaccination on Day 28, all ilunocitinib treated animals in this study would clinically be considered fully immunized against the ML core vaccines (CAV-2, CDV, CPV) as tested on Day 60 [15]. Despite the outbreak of disease during the time of vaccination all but one animal that had an adequate titer on Day 60 following primary vaccination on Day 28, also had an adequate titer response to ML core vaccines on Day 88 following booster vaccination on Day 60. Results are shown in Table 4; Fig. 7.

**Table 4:** Titer results

Group	Virus	Number (n) and Percent (%) of animals at or above threshold		
		Day 60 n (%)	Day 88 n (%)	Day 116 n (%)
1 (0X) *n* = 8	CAV-2	8 (100)	8 (100)	NA
	CDV	8 (100)	8 (100)	NA
	CPiV	0 (0)	6 (75)	NA
	CPV	8 (100)	8 (100)	NA
	Rabies	NA	8 (100)	7 (87.5)
2 (3X) *n* = 6	CAV-2	6 (100)	6 (100)	NA
	CDV	6 (100)	5 (83)	NA
	CPiV	2 (33)	4 (67)	NA
	CPV	6 (100)	6 (100)	NA
	Rabies	NA	2 (33)	5 (83)

#### Inactivated vaccine

**Fig. 8:**
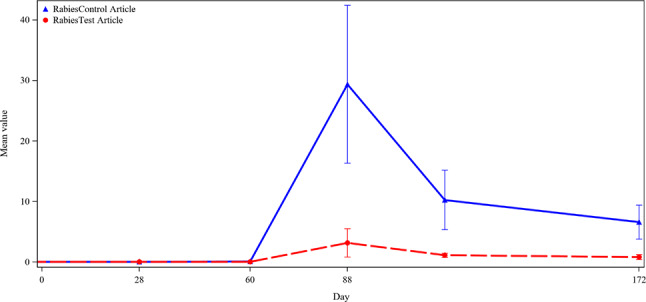
Titer Results for Rabies Virus. Legend: Mean Titer results Rabies virus throughout the study

The antibody response to primary rabies virus (RV) vaccination (administered on Day 60) met the threshold for immunity on Day 88 in two of six treated dogs in the ilunocitinib group (Fig. 8). The four low responding dogs were the same dogs described above as having insufficient Th cell counts. The two dogs with Th cells within the reference range were the successful responders to primary rabies vaccination. In this study there is a correlation between health status as assessed by Th cell counts and rabies vaccine response. All dogs in the placebo group met the titer threshold at the Day 88 timepoint. Although treatment with ilunocitinib ended on Day 88, an additional titer for rabies only was measured on Day 116, at which time all ilunocitinib treated dogs, except 735, had rabies titers above threshold. A single dog from the control group also had a titer below threshold at this timepoint.

## Discussion

The original objective of this study was to determine if treatment with ilunocitinib at 3X the recommended dose for an extended duration impacted the response to ML or inactivated vaccines in healthy, barrier-raised, vaccine-naive, sero-negative, juvenile, purpose-bred research dogs. However, due to an outbreak of unanticipated infectious diseases (including coccidiosis and ICH) the study was confounded from its original intent. Molecular diagnostics confirmed the presence of CAV-1 in the fixed liver tissue of dog 495 confirming CAV-1 as the cause of ICH in this dog. While the source of the CAV-1 remains unclear, the vaccine virus has been ruled out as the cause of ICH in this dog by the described sequencing data. Other potential sources of CAV-1 include a CAV-1 carrier of latent virus or indirect exposure to wild type virus, despite biosecurity measures.

Many factors influence the serological response to both live and inactivated vaccines including age, breed, stress, altered immunocompetence, concurrent disease, biologic variation, and nutritional status [16–18]. Vaccine labels warn to only vaccinate healthy, non-parasitized dogs. Due to the confounding factors of parasitism and infectious diseases that occurred during this study a clear conclusion of drug effect on response to vaccination cannot be made as intended. Additionally, it is important to note these study animals were unhealthy and not representative of the typical clinical patient population. Despite all these factors, the results are important to analyze as useful data to add to the growing knowledge on the use of JAK inhibitors in dogs.

Given the well characterized roles of the JAK pathway in immune cells and the importance of immune cells in mediating vaccine responses, IMT was planned as an original component of the study. The most noticeable change was evident in the CD4 + T Helper lymphocytes (Th cells) which significantly decreased at Day 28 in some ilunocitinib and placebo treated dogs, Fig. 6. Due to the comparatively little change in CD8 + Cytotoxic T lymphocytes (Fig. 6), the decrease in total lymphocytes is likely due to the reduction in Th cells. The decrease in the CD4 + Th cell population in dogs from both the placebo (6/8) and the ilunocitinib (5/8) groups occurred prior (between Day − 7 and 28) to the time period with the most severe clinical disease and administration of the first ML vaccine. JAK enzymes are expressed on a wide variety of cell types, including both CD4 + Th and CD8 + cells [6, 19, 20]. Based on this, if the Th cell decrease in this study were solely treatment-related, an effect on CD8 + T cells would have been expected but did not occur. The presence of similar reduction in Th cells between treatment and placebo groups in this study also suggests the change was not solely treatment related. Additionally, the pivotal margin of safety study completed for ilunocitinib showed no dramatic drop in Th cells in dogs treated up to five times the recommended dose for 6 months and confirms no test article related effect on any IMT parameter evaluated [21]. Coccidiosis in other species has been associated with lymphopenia and T cell depletion because of T cells infiltrating the gut tissue in response to infection [22–24]. This study supports the hypothesis that the same phenotype occurs in dogs with coccidiosis. An observed decrease in Th cell counts corresponded to disease severity in the ilunocitinib group and greater impact on titer levels compared to the placebo group. Dog 495 developed hematemesis on day 21 and continued to be housed in the same facility as the other study dogs until day 54. At such time the dog was removed from the study and subsequently diagnosed with ICH. Infection with CAV-1 in dog 495 was not definitively identified until PCR testing was performed post study completion, and therefore it is unknown to what extent other dogs may have also been infected. Because CAV-1 is easily transmitted between unvaccinated dogs [25, 26], it is plausible that other dogs may have been infected with CAV-1 by the time the samples for IMT were taken on day 28. Without the data to understand the incidence and timing of potential CAV-1 infection across all dogs on study, it is impossible to know to what extent CAV-1 infection in addition to coccidiosis may have had on the IMT results and subsequent response to vaccination.

The ML primary vaccine given at Day 28 elicited a response meeting the threshold for protection in all dogs (control and JAK inhibitor treated) as measured by titers on Day 60. The achievement of reaching threshold at that timepoint was surprisingly impressive given the presence of confounding concurrent infections. Clinical signs of disease were most prevalent between Days 35–60. This is also the time when the dogs had diminished health as assessed by reduced food intake and body condition. There was not an immunophenotyping sample timepoint taken between Day 28 and Day 88, but the fact that cell counts had not returned to baseline levels at the Day 88 timepoint suggests that levels were most likely decreased at Day 60 therefore hindering the dogs’ ability to respond to vaccination at this timepoint (Figs. 5 and 6). The ML booster and the primary Rabies vaccine were administered on Day 60 when the dogs were recovering from GI disease. Due to the compromised health status, as demonstrated by decreased CD4 + Th cells and body condition in treated animals during the time of ML booster and RV vaccination on Day 60, it is not surprising that antibody responses did not meet the established criteria for success on Day 88 in some dogs (Table 4). This finding agrees with a recent study where body condition was shown to be a statistically significant variable in the probability of Rabies vaccine seroconversion [27]. Overall, the achievement of threshold titers at Day 28 by all dogs and the relative success of the treated dogs on Day 88 combined with the associated clinical data suggest that mechanistic influence of drug had more to do with the dog’s response to infections indirectly affecting the ability to respond to the vaccines as opposed to a direct effect on the vaccine response itself.

It is well accepted that vaccine responses are hindered with disease; a specific study demonstrated dogs under 1 year old with endoparasites had significantly lower titers 28 days after rabies vaccination compared to healthy controls [28]. This parallels what happened in the current study with vaccination of unhealthy, young dogs achieving suboptimal titer response 28 days following rabies vaccination. In humans, CD4 Th cells are viewed as essential for an adequate immune response against rabies [29]. Although this study did not have an IMT datapoint within the day 35–60 window where clinical disease was highest (Fig. 2), nearly preceding is the day 28 datapoint where Th cell lymphopenia was observed. It is probable that the initial suboptimal response to the rabies vaccine is related to the decline in Th cells, and in fact the animals with the lowest Th cell counts correlate with lower titer responses. These results are not unexpected as Th cells are especially required to mount an immune response to inactivated vaccines, as compared to ML vaccines [30].

Just as immune system changes mediated by disease can interfere with vaccine responses, so can immune system changes mediated by other mechanisms. It therefore follows that immune modulatory drugs may interfere with vaccinations. Although we are unable to conclusively assess JAK inhibition on ML and rabies vaccination in dogs within the current study, it would be expected that withholding drug around the time of vaccination may be appropriate for otherwise unhealthy or immunosuppressed patients, based on the mechanism of action of JAK inhibitors and consistency with currently approved drugs in the JAK inhibitor class.

## Conclusion

JAK inhibitors are well-known for their success in modulating pathologic inflammatory responses including AD in dogs. Dogs in this study were treated with 3X the recommended dose of an investigational drug. As would have been expected during an outbreak of infectious disease, treated animals had increased morbidity and mortality compared to placebo treated controls. In the face of a worst-case scenario of concurrent disease-induced poor health and 3X drug treatment for an extended duration, rabies vaccine titers failed to reach target levels according to the original study criteria on Day 88, but did have an improved response by Day 116. The most surprising result was that despite the worst-case scenario that manifested during this study, all dogs in both treatment groups achieved the target titer level after primary ML vaccination on Day 60. Overall, this study confirms that humoral vaccine responses in sick animals may be suboptimal, and high doses of JAK inhibitors may result in immune suppression resulting in more severe clinical signs associated with infectious diseases. It also confirms vaccines should not be given to unhealthy animals or animals who are potentially immunocompromised. Due to the confounding variables of infectious disease in the current study, it is impossible to draw a definitive conclusion as per the original study intent. Future studies should evaluate the impact of ilunocitinib on response to vaccination in dogs free from infectious diseases and with adequate Th cell counts at the time of vaccination.

## Electronic supplementary material

Below is the link to the electronic supplementary material.

Supplementary Material 1

Supplementary Material 2

## Data Availability

Data is provided within the manuscript.

## Abbreviations

JAK: Janus-Kinase
TYK2: Tyrosine Kinase 2
AD: Allergic and Atopic Dermatitis
ML: Modified Live
3X: Three Times
CDV: Canine Distemper Virus
CPV: Canine Parvovirus
CAV-2: Canine Adenovirus-2
CPiV: Canine Parainfluenza Virus
GLP: Good Laboratory Practice
ABSL2: Animal Biosafety Level 2
CBC: Complete Blood Count
IMT: Immunophenotyping
RFFIT: Rapid Fluorescent Focus Inhibition Test
PCR: Polymerase Chain Reaction
FFPE: Formalin-Fixed Paraffin Embedded
GI: Gastrointestinal
ICH: Infection Canine Hepatitis
ACTB: Canine Actin Beta Gene
USDA: United States Department of Agriculture
RV: Rabies Virus
Th cells: T Helper Lymphocytes
