# Supplementary figures and images for: Response to primary canine core vaccination in 10-month-old seronegative dogs treated with three times the recommended therapeutic dose of Ilunocitinib tablets (Zenrelia™)
Source: BMC Vet Res. 2025 Jul 14;21:461. doi: 10.1186/s12917-025-04896-5 (PMC12257700; doi:10.1186/s12917-025-04896-5)

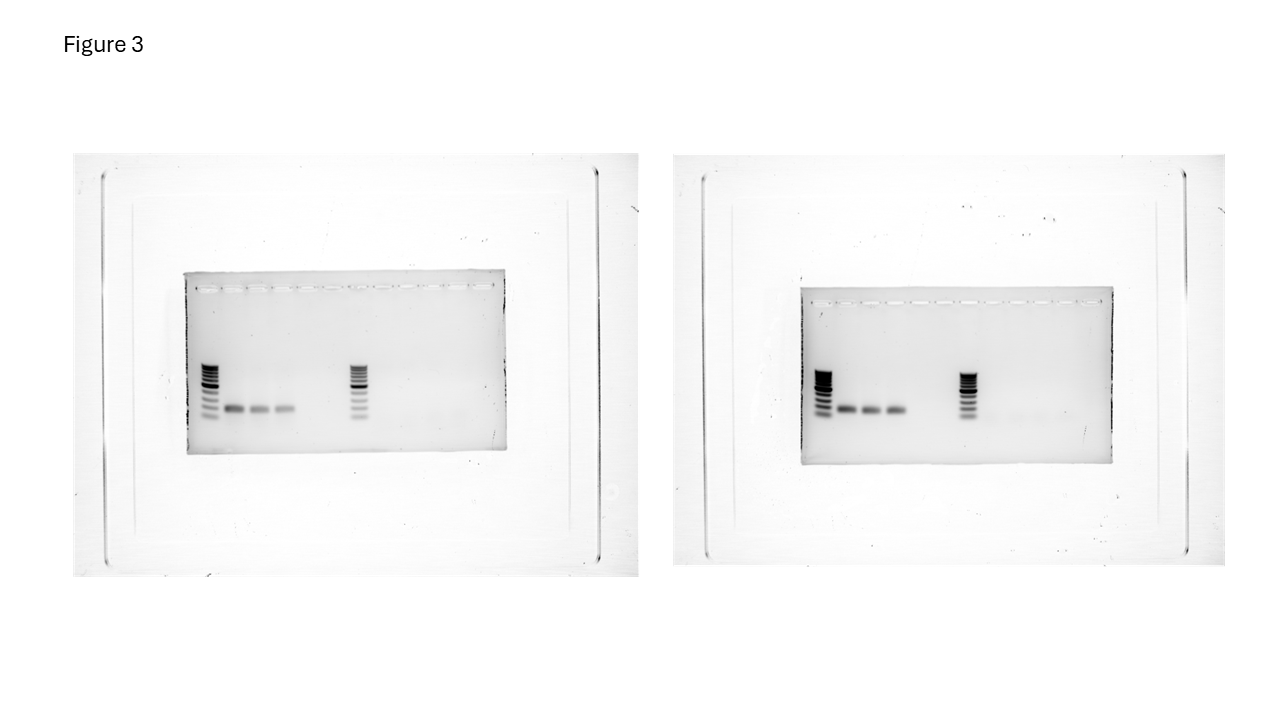

Supplement: Supplementary file 1 — Supplementary Material 1 [file 12917_2025_4896_MOESM1_ESM.tif]

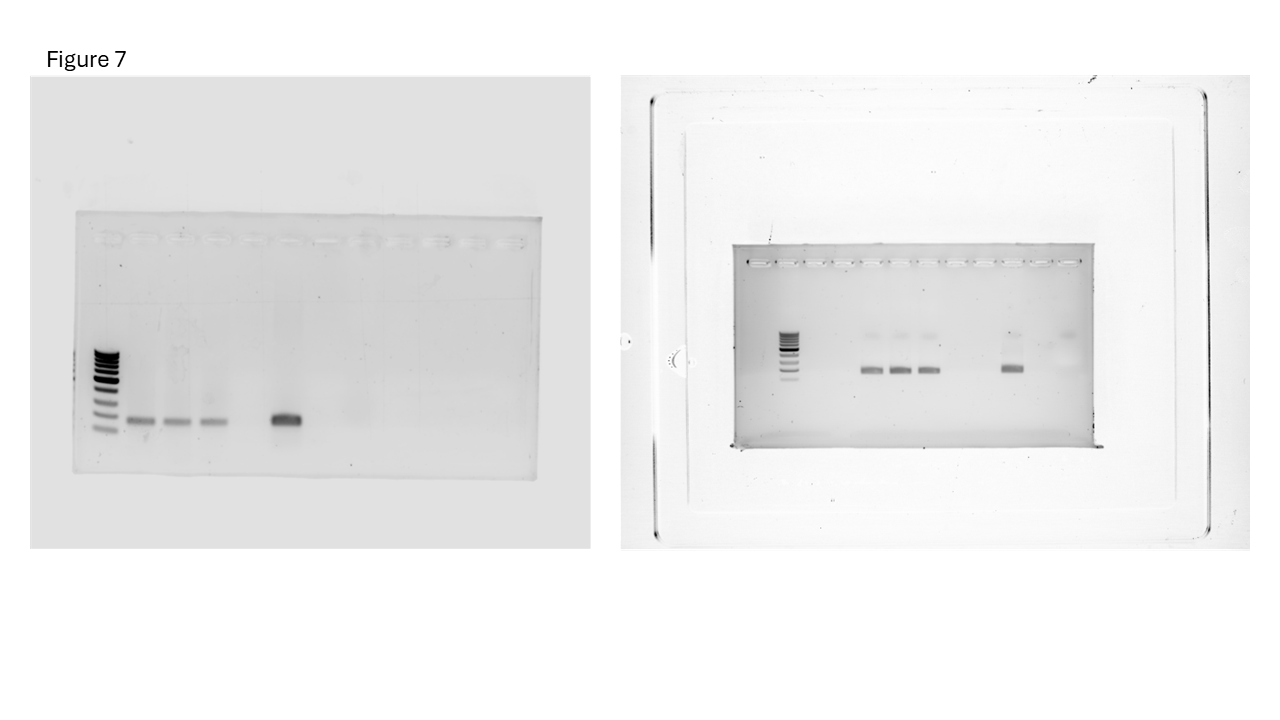

Supplement: Supplementary file 2 — Supplementary Material 2 [file 12917_2025_4896_MOESM2_ESM.tif]
